# Supplementary material for: A Critical View of the Application of the APEX Software (Aqueous Photochemistry of Environmentally-Occurring Xenobiotics) to Predict Photoreaction Kinetics in Surface Freshwaters
Source: Molecules. 2019 Dec 18;25(1):9. doi: 10.3390/molecules25010009 (PMC7017383; doi:10.3390/molecules25010009)
Supplement: Supplementary file 1 [file molecules-25-00009-s001.zip › 3.2.4_gcc-4.4.0.zip!/3.2.4_gcc-4.4.0/3.2.4_gcc-4.4.0/doc/octave/html/interpreter/Anonymous-Functions.html]

Anonymous Functions - Untitled


Next: Inline Functions,
Previous: Function Handles,
Up: Function Handles Inline Functions and Anonymous Functions


---

#### 11.9.2 Anonymous Functions

Anonymous functions are defined using the syntax

```
     @(argument-list) expression
```

Any variables that are not found in the argument list are inherited from
the enclosing scope. Anonymous functions are useful for creating simple
unnamed functions from expressions or for wrapping calls to other
functions to adapt them for use by functions like `quad`. For
example,

```
     f = @(x) x.^2;
     quad (f, 0, 10)
         => 333.33
```

creates a simple unnamed function from the expression `x.^2` and
passes it to `quad`,

```
     quad (@(x) sin (x), 0, pi)
         => 2
```

wraps another function, and

```
     a = 1;
     b = 2;
     quad (@(x) betainc (x, a, b), 0, 0.4)
         => 0.13867
```

adapts a function with several parameters to the form required by
`quad`. In this example, the values of a and b that
are passed to `betainc` are inherited from the current
environment.
